# Evaluation of 3′,4′-Di-*O*-acetyl-*cis*-khellactone as a Putative Antagonist of PPARγ Using Experimental and Computational Modeling

**DOI:** 10.3390/biom16050724

**Published:** 2026-05-14

**Authors:** Elix Alberto Domínguez-Mendoza, Fernando Daniel Prieto-Martínez, Yelzyn Galván-Ciprés, Eleuterio Burgueño-Tapia, Cynthia Ordaz-Pichardo

**Affiliations:** 1 Departamento de Sistemas Biológicos, Universidad Autónoma Metropolitana-X, Ciudad de México 04960, Mexico; 2 Instituto de Ciencias Físicas, Universidad Nacional Autónoma de México, Av. Universidad s/n, Col. Chamilpa, Cuernavaca 62210, Morelos, Mexico; 3 Departamento de Química Orgánica, Escuela Nacional de Ciencias Biológicas, Instituto Politécnico Nacional, Ciudad de México 07230, Mexico; ygalvanc2200@alumno.ipn.mx (Y.G.-C.); eleuteriobt@gmail.com (E.B.-T.); 4 Escuela Nacional de Medicina y Homeopatía, Instituto Politécnico Nacional, Ciudad de México 07230, Mexico

**Keywords:** antiobesity, molecular dynamics, molecular modeling, NAFLD, obesity, PPARγ

## Abstract

Obesity and Non-Alcoholic Fatty Liver Disease (NAFLD) are closely linked and constitute a growing public health concern. In this work, we evaluated the antiobesity effect of an enantiomerically enriched mixture of 3′,4′-di-*O*-acetyl-*cis*-khellactone (DOAcK), a natural product derivative obtained by asymmetric synthesis. This molecule is a derivative of praeruptorin, a major component found in *Arracacia tolucensis* as well as many other species from the *Apiaceae* family. A comprehensive evaluation of DOAcK was conducted using both experimental and theoretical methods. DOAcK showed significant effects in animal models, with additional evidence of diminished expression of PPARγ. Finally, we conducted molecular modeling to elucidate the putative interaction between DOAcK and PPARγ, uncovering the significant role of the so-called Ω-loop, near the ligand binding domain. In summary, these positive findings of DOAcK demonstrate that the use of this natural product could be helpful in preventing NAFLD and obesity.

## 1. Introduction

Obesity is characterized by excessive fat accumulation that poses a major risk to an individual’s health [1]. The number of people with obesity has increased in recent years, and according to the Organization for Economic Co-operation and Development (OECD) [2], countries such as the United States of America, Mexico, New Zealand, the United Kingdom, and Hungary have the highest prevalence of obese adults. Poor dietary habits and a sedentary lifestyle contribute to the spread of this condition. Despite preventive efforts such as social programs and approved drugs such as Orlistat, the incidence of obesity continues to rise. Diabetes, hypertension, heart stroke and Non-Alcoholic Fatty Liver Disease (NAFLD) are diseases commonly associated with obesity [3]. NAFLD prevalence is higher in diabetic and obese people and there is still not an effective pharmacotherapy to decrease the damage generated by this disease [4]. An increase in lipids such as cholesterol, alongside insulin resistance and low-grade inflammation, are hallmarks of both obesity and NAFLD.

The primary role of white adipose tissue (WAT) is the storage of circulating free fatty acids (FFAs), such as triglycerides. Additionally, WAT exhibits endocrine function by releasing pro-inflammatory cytokines that influence cell functions.

Peroxisome proliferator-activated receptor gamma (PPARγ) belongs to the nuclear receptor family, which regulates energy homeostasis, both increasing and decreasing multiple gene expression (pro-inflammatory cytokines and energy control genes) [5]. The therapeutic potential of PPARγ has been explored as a subject of many bioactive compounds like barbituric acid derivatives [6], phenylpropanoic acids [7], and harpagosides [8] which were proposed as potential candidates for the treatment of diabetes and obesity. Furthermore, pioglitazone, a known PPARγ agonist, has been used to treat NAFLD [9]. In lieu of this, we have previously reported the antidiabetic activity of an enriched enantiomeric (EE) mixture of 3′,4′-Di-*O*-acetyl-*cis*-khellactone (DOAcK; *R*,*R*, 70%) [10]. We hypothesized that the observed effect was mediated by modulation of PPARγ. Moreover, this compound exhibited no evident signs of toxicity such as non-mutagenic according to the AMES test, non-genotoxic effect according to the micronuclei test and its DL_50_ placed it in category 5, according to the OECD TG423 test [10]. Therefore, the aim of this study was to evaluate this natural product derivative in a high-fat diet-induced obesity model to determine its therapeutic potential for the treatment of NAFLD.

## 2. Materials and Methods

### 2.1. Animals

A total of 32 C57BL/6 mice (25.0 ± 5.0 g) were obtained from an institutional animal facility and randomly divided into four groups (n = 8 per group). The animals were housed under controlled conditions at 25 °C with a 12 h light/dark cycle and provided with standard food and water ad libitum. Following a one-week acclimatization period, mice were identified using tail tags. The experimental protocol was approved by the Institutional Review Board of Instituto Politécnico Nacional (ENMH-CB-141-2015, 30 September 2015) and following the regulatory norms of the Mexican government (NOM-062-ZOO-1999).

### 2.2. Obesity Induced by the High-Fat Diet Model

The first week was for adaptation, in which only a standard diet was provided, followed by six weeks where the animals were only fed with a high-fat diet (HFD) and underwent no respective treatment. Starting at the seventh week, and for the remainder of the experiment (10 weeks), mice were administered with the different treatments using an intragastric gavage. The dosage for DOAcK was 15 mg/kg.

The animals were provided with the following diets, chosen at random:

- Group 1 Control mice were fed a standard diet containing 13.1% fat, 59.8% carbohydrates, and 27% protein.
- Group 2 HFD Control mice were fed a high-fat diet containing 60.3% fat, 21.3% carbohydrates, and 18.4% protein.
- Group 3 HFD + Orlistat mice were fed a high-fat diet + Orlistat (Orlistat was orally administered at 5.14 mg/kg body weight).
- Group 4 HFD + DOAcK mice were fed a high-fat diet + DOAcK (DOAcK was orally administered at 15 mg/kg body weight).

### 2.3. Body Weight and Biochemical Parameters

Experimental body weight was measured once a week at the same time every morning. After ten weeks of the experimental protocol, the mice fasted overnight and fasting blood glucose was then measured with a manual glucometer (Accucheck ActiveBlood samples were collected from the tail tip prior to sacrifice). Euthanasia was performed by CO_2_ inhalation using a dedicated chamber, in accordance with institutional guidelines. Tissue samples were collected in separate vials containing RNAlater^®^ or formaldehyde for subsequent analyses.

### 2.4. Histological Analysis

Liver tissue samples were collected and immediately fixed in 10% neutral-buffered formalin for 24 h at room temperature. Fixed samples were subsequently dehydrated through a graded ethanol series, cleared in xylene, and embedded in paraffin wax. Sections of 4 μm thickness were prepared using a rotary microtome and mounted onto glass slides. Prior to staining, the sections were deparaffinized in xylene and rehydrated through descending ethanol concentrations to distilled water. Hematoxylin and eosin (H&E) staining was performed to assess general tissue morphology. Slides were examined under a light microscope, and images were captured using a digital camera system for subsequent quantitative and qualitative analysis. All procedures were conducted in accordance with institutional guidelines for the handling and processing of biological tissues.

### 2.5. RNA Extraction and Polymerase Chain Reaction

RNA extraction from adipose and liver tissues was performed using the Trizol^®^ protocol (Sigma-Aldrich, Mexico). RNA was quantified using the NanoDrop spectrophotometer (Thermo Fisher Scientific, Mexico). cDNA was synthesized from RNA using reverse transcriptase. Briefly, RNA 0.5 µg was treated with dT primer, DNTPs and water to accomplish a 12 µL volume. This mix was set to 65 °C for 5 min and then the following reactants were added: DTT, RNAaseOUT and Superscript II, which were incubated at 42 °C for 50 min. cDNA strains were used to evaluate changes in the RNAm by final point PCR. DNA amplification products were carried out in agarose gel electrophoresis at 2%, and ethidium bromide was applied as dye. Thermocycler conditions were 30 s at 95 °C for elongation, 35 cycles of 5 s denaturation at 95 °C, 34 s extension at 60 °C, and 10 min at 4 °C to stop the reaction. Primer sequences are shown in Table S1.

### 2.6. Molecular Modeling

Due to the nature of PPARγ, an overarching difficulty is the assessment of the putative binding of ligands, as discussed elsewhere [11]. To this end, we conducted a comprehensive characterization using several methods, starting with an ensemble of PPARγ structures (PDB: ID 4JAZ, 5YCP, 6C5Q, 6C5T, and 7WOX). We conducted molecular docking and dynamics and enhanced sampling simulations using well-tempered metadynamics and adaptive sampling to elucidate free energy surfaces using Markov state models (MSM).

### 2.7. Molecular Docking

PLANTS software (v 1.2) [12] was used for docking calculations; all PDB complexes were stripped from ligands and water molecules. Hydrogens and missing residues were added using PDBfixer 1.8 (https://github.com/openmm/pdbfixer, accessed on 7 April 2026). The complete structure was then processed with SPORES 1.3 [13]. Marvin was used for drawing the structure of DOAcK, Marvin 22.7, 2022, ChemAxon (http://www.chemaxon.com, accessed on 7 April 2026). To account for the EE mixture, molecular modeling was done just with the *R*,*R* isomer. The obtained 2D structure was then converted to a 3D conformer with Openbabel 3.1.0 [14]. Geometry optimization and charge calculation were carried out with MOPAC 22.0.6 (MOPAC2016, James J. P. Stewart, Stewart Computational Chemistry, Colorado Springs, CO, USA, HTTP://OpenMOPAC.net (2016)), using the PM6-D3H4X method. The search space was defined by a sphere around the co-crystal ligand in each structure, with a radius of 10 Å; clustering of poses was kept at default values.

### 2.8. Binding Mode Assessment

To prune molecular docking results, we used a previously described protocol [15] which uses metadynamics to enhance sampling and get an estimate of the viability and stability of a given pose. It has been suggested that prior equilibration of water molecules greatly improves the results. Thus, we carried out this step using grand, a Python (v 3.9) library that uses grand-canonical Monte Carlo to hydrate the binding site [16]. Complexes were prepared using Ambertools 22; parametrization included AMBER14SB for protein atoms, TIP3P as the water model, and ligand atoms used GAFF 2. Simulations were run under OpenMM 8.0, using the middle Langevin integrator with a timestep of 4 ps with the Monte Carlo barostat. Open Binding Pose Metadynamic (OBPMD) scripts were run using default parameters, i.e., 10 replicas for each pose, using cherry-picked binding modes selected based on molecular interactions and docking scores. Promising binding modes were selected based on CompScore (henceforth referenced as OBPMD score), as a heuristic criterion.

After identifying the most promising candidate, additional simulations using well-tempered metadynamics were conducted for 500 ns. These simulations were performed using the Desmond engine [17] within the academic version of Maestro 22.1. For consistency, parameters were kept the same; i.e., AMBER14SB for protein TIP3P for water and GAFF 2 for the ligand. Parameters were assigned using Viparr 4.0, as illustrated elsewhere [18], using the ligand’s center of mass RMSD and distance from anchoring residues as collective variables. Gaussian functions with a height of 0.03 kcal/mol and width of 0.15 Å (for both cases) were deposited at a rate of 500 steps with a bias factor of 4.0. Ensemble conditions were enforced with the Langevin thermostat and barostat with coupling values of 0.02 and 1.0 ps, respectively. The integration timestep was kept at 2 and 4 fs using the RESPA integrator.

Following binding mode analysis, we conducted adaptive sampling with classical MD simulations using Desmond. Based on prior results, and considering that higher precision of sidechain movement was needed, we selected 4JAZ results and reparametrized complexes with an AMBER19SB force field, OPC water model and TIP4PEW parameters for ions [19]. Similar conditions to those from metadynamics runs were used.

The protocol followed common practices in MSM [20]. In our case, we conducted an initial simulation of 1 µs, with shorter replicas between 250 and 750 ns. To obtain further diversity in our sampling, we conducted short metadynamics runs; selected snapshots (from high energy regions) were minimized and equilibrated for 100 ns prior to production. These were used to build an initial MSM, based on inverse distances between pocket heavy atoms and ligand heteroatoms, using PyEMMA (v.2.5.11). Using time-independent component analysis (TICA), clustering and spectral analysis, additional seeds were selected based on density and microstate membership. With these, over a hundred trajectories were obtained accounting for ~10 µs.

Finally, we conducted well-tempered metadynamics with pocket solvation and AMBER19SB. Conditions were similar to the previously described runs; yet, for these, production times were 500 ns.

### 2.9. Statistical Analysis

Data were analyzed using one- or two-way ANOVA followed by Dunnett’s post hoc test, as indicated in each graph; Barlett’s test was used to verify homoscedasticity. Statistical significance was defined as *p* < 0.05.

## 3. Results

### 3.1. In Vivo Activity

**Figure 1:**
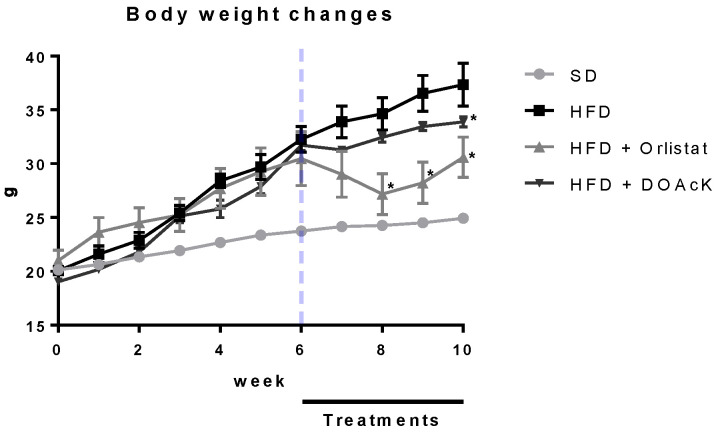
Effect of DOAcK on body weight of mice fed with HFD. * Statistically significant difference versus the obese group by multiple comparison Dunnett test. Treatments were started at week 6 of HFD. (n = 8, mean ± SEM, *p* < 0.05).

**Figure 2:**
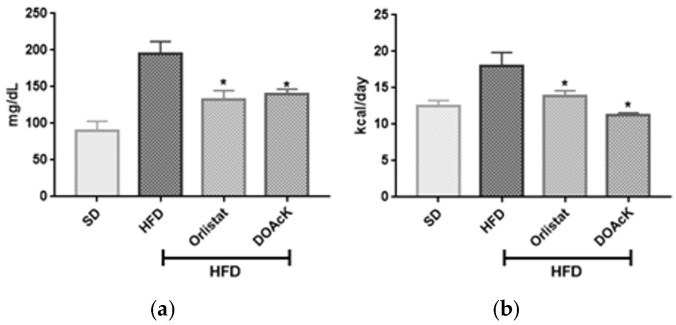
Effect of DOAcK on (**a**) fasting blood glucose and (**b**) kcal consumed daily. * Statistically significant difference versus obese group by multiple comparison Dunnett test. Treatment was started at week 6 of HFD. (n = 8, mean ± SEM, *p* < 0.05).

Administration of DOAcK in a high-fat diet model showed a decrease of 33.88 ± 0.48 g versus Orlistat 30.59 ± 1.87 g. These values had statistical significance at week 10 when compared with the HFD group (37.34 ± 1.97) (Figure 1). Figure 2 shows fasting blood glucose (FBG) levels and caloric intake across the different groups. DOAcK significantly reduced FBG compared with the HFD group (140.3 vs. 195.3 mg/dL). Interestingly, the group treated with DOAcK decreased the kcal intake (11.3 kcal) in contrast with the HFD group (18.0 kcal).

To determine whether the decrease in body weight affected key organs, the mass of the liver and other relevant organs was measured. There was no significant difference between HFD versus DOAcK groups in fat pad mass. However, the spleen, liver, kidney, and pancreas showed decreased mass in the DOAcK-treated group compared with the HFD group (Table 1).

**Figure 3:**
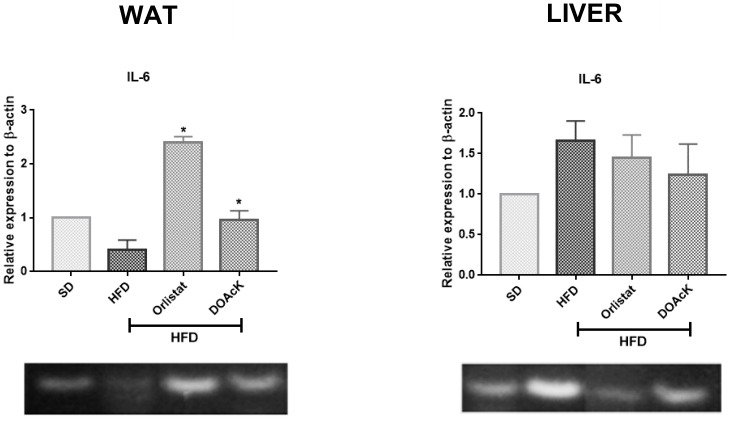
Effect of DOAcK on relative expression of IL-6 in WAT and the liver. * Statistically significant difference versus HFD group by multiple comparison Dunnett test. (n = 3, mean ± SEM, *p* < 0.05). The original electrophoresis images can be found in the Supplementary Materials.

**Figure 4:**
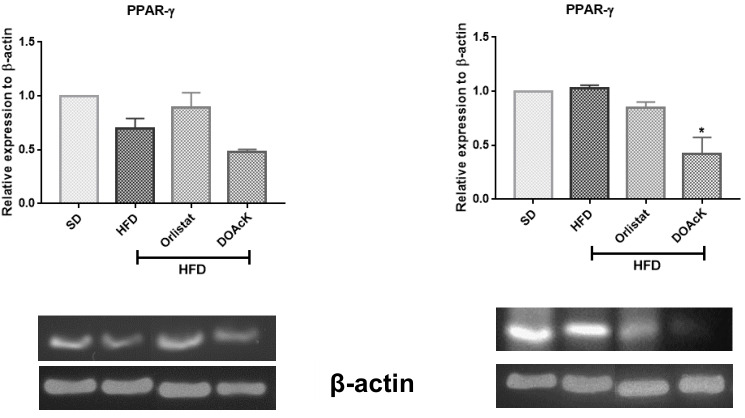
Effect of DOAcK on relative expression of PPAR-Y in WAT and the liver. * Statistically significant difference versus HFD group by multiple comparison Dunnett test. (n = 3, mean ± SEM, *p* < 0.05). The original electrophoresis images can be found in the Supplementary Materials.

In addition, molecular analysis showed that DOAcK exerts a modulatory effect on IL-6 and PPARγ expression (Figure 3 and Figure 4). In WAT, DOAcK restored IL-6 expression, while also reducing PPARγ expression in the liver as compared to the HFD group. It is important to mention that endpoint PCR was employed as a semi-quantitative approach to evaluate relative expression changes in PPARγ and IL-6. The results are preliminary and more assays are needed to support these results.

### 3.2. In Silico Assays

Docking was performed to determine the possible interaction between PPARγ and DOAcK. To this end, we conducted ensemble docking to better assess pocket variations, as these subtle changes determine the interaction of agonists and antagonists at molecular level.

Pruning of docking poses is paramount during the early stages of binding assessment, and for this work, we focused on consensus to drive decision making. Interestingly, docking scores for DOAcK were quite consistent within the ensemble. Notably, the gem-dimethyl group presents multiple hydrophobic interactions; this aliphatic group has been shown to be widely useful in medicinal chemistry to develop bioactive molecules with improving potency, selectivity, and pharmacokinetic properties [21].

In contrast, the results from PDBID 5YCP show DOAcK within the thiazolidinediones site [22]. This, in addition with the observed contacts with C285 in most poses, may suggest agonist activity [23]. However, this may not be the case within a dynamic context, as proven recently [24]. For instance, the pose obtained from PDB ID 7WOX showed considerable promise, as contacts with G284 were maintained.

**Figure 5:**
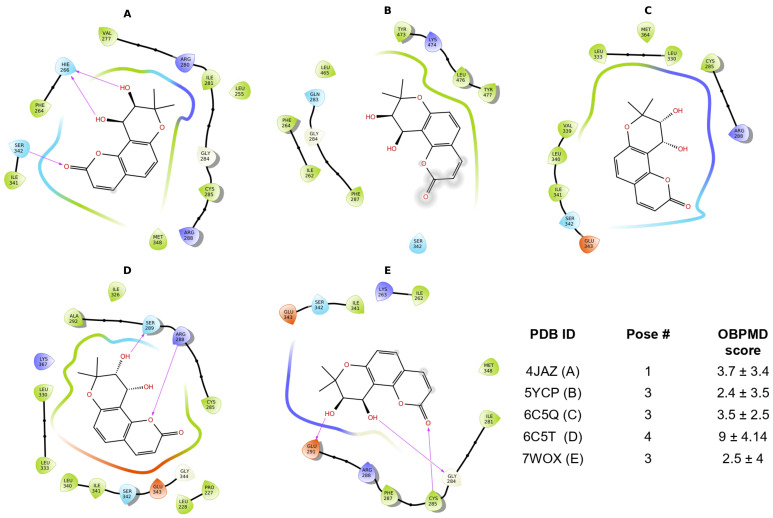
Selected binding modes obtained from the protein ensemble of PPARγ and DOAcK.

Therefore, a series of short metadynamics runs were done; Figure 5 presents the selected poses and their scoring. It can be seen that poses had comparable scoring values; however, deviation seemed very significant (see Supplementary Information, Figure S1). This is to be expected, as short well-tempered metadynamics runs tend to produce noisy results. Nevertheless, the fact that all complexes exhibited this behavior also suggests that the initial binding modes are metastable.

**Figure 6:**
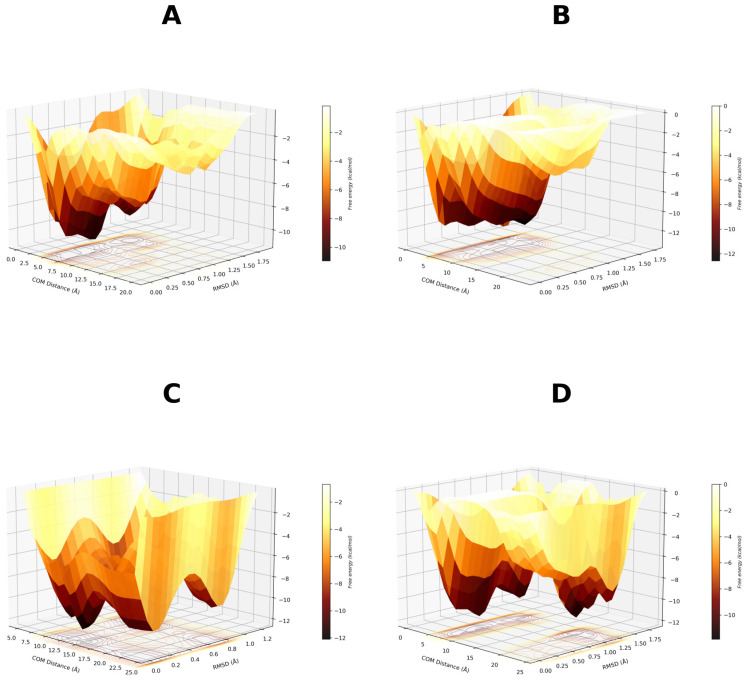
Free energy surfaces obtained for selected binding modes using well-tempered metadynamics. (**A**) 4JAZ-DOAcK. (**B**) 6C5T-DOAcK. (**C**) 7WOX-DOAcK with GCMC solvation. (**D**) 7WOX-DOAcK without GCMC solvation.

To further investigate this hypothesis, the 4JAZ, 6C5T, and 7WOX structures were selected for additional well-tempered metadynamics simulations. The rationale behind this approach was twofold: to further explore the putative metastability of the complexes and to determine the role of pocket solvation in ligand binding. In this scenario, 4JAZ complex with GCMC hydration was selected due to its neutral scoring, and 6C5T complex (worst scoring) without hydration. Finally, 7WOX emerged as the best-scoring candidate in both the GCMC and non-GCMC iterations. The results are presented in Figure 6.

Based on FES delimitation, we confirmed binding mode metastability, with 7WOX-DOAcK complex being the most promising. Of note, both 6C5T and 7WOX are co-crystallized with PPARγ antagonists. As stated earlier, pocket conformation of 6C5T seems unfavorable for DOAcK, as there is a notable misalignment of pharmacophoric elements when compared to SR11023 (see Supplementary Information; Figure S2). Conversely, results from the 7WOX complex show a higher affinity due to a well-defined local minimum in a metastable basin. Moreover, this trait seems related to pocket solvation due to high energy barriers seen on the standard protocol.

At this stage, it does seem that DOAcK has an affinity for the ligand binding domain of PPARγ, but we still had to elucidate if the apparent preference would be toward inactivation or at least partial modulation. Thus, we selected the 4JAZ-DOAcK complex with GCMC solvation and carried out an adaptive sampling protocol. This technique is very useful and robust for Markov state modeling. However, our intended goal was not MSM construction per se. Considering our resources and overall noise in the simulations, we were not interested in kinetic validation or phenomena; we just tested for alternative contacts from the initial pose.

**Figure 7:**
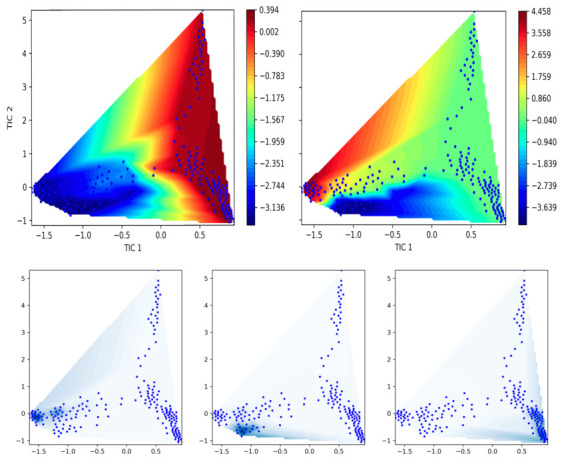
Spectral analysis of free energy surface, using TICA. Two main processes are identified with three basins.

For our analysis, we chose inverse distances between ligand heteroatoms and protein residues within 1 nm of DOAcK, which in turn were used for TICA and FES projection, considering implied timescale (ITS) validation (see Supplementary Information; Figure S3); we chose a lag time of 1 ns for TICs and the MSM was built with a lag of 10 ns. Additionally, ITS analysis also suggests two slow processes, confirmed by spectral analysis of eigenvalues (Figure 7 and Figure S4).

With this model, FES projection was made, confirming that the obtained sample is robust enough for our intended purpose. Nevertheless, blank regions persist at the junction which seems to characterize macrostate transition. Still, we consider that sampled energy basins may belong to alternative binding modes worth exploring.

**Figure 8:**
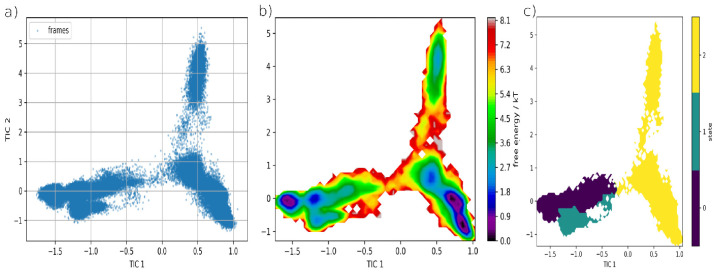
Obtained sample projected on TIC space as simulation frames (**a**) Free energy surface (**b**) and macrostates delimited by PCCA (**c**).

Hence, we chose to continue with three macrostates based on basin presence and ITS. Figure 8 shows frame projection on TIC space, the corresponding free energy surface and Robust Perron Cluster Analysis (PCCA) based on three macrostates.

**Figure 9:**
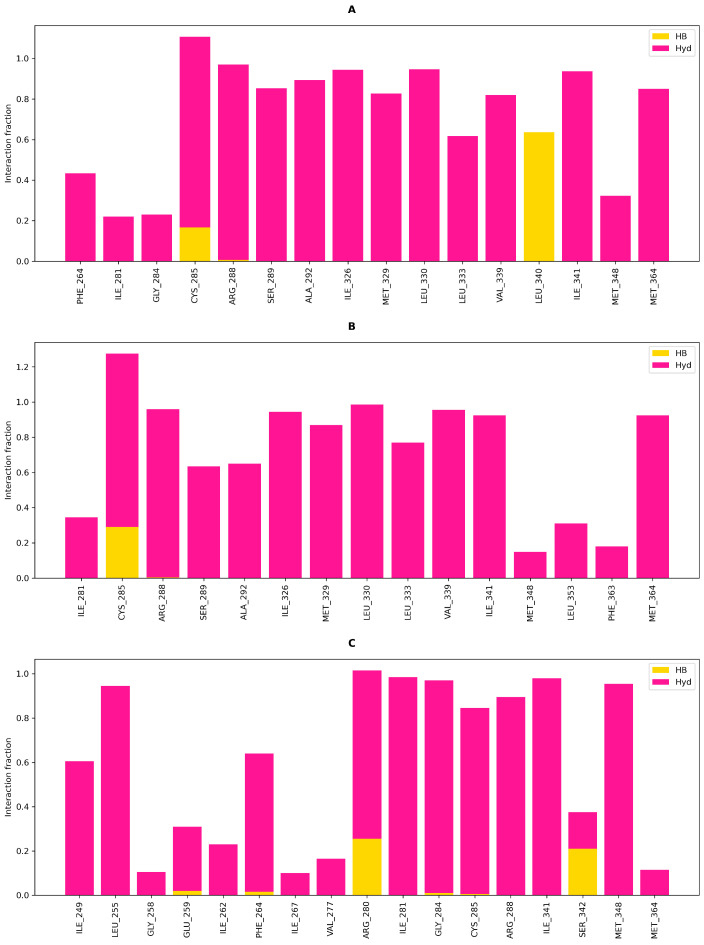
Contact analysis for the macrostates obtained from adaptive sampling and PCCA. Interaction fraction for macrostates 1, 2 & 3 (**A**, **B** & **C**; respectively).

Macrostate 1 shows a clear basin with a local minimum, while macrostate 2 belongs to a high energy ensemble for the most part. It is possible that said macrostate is partly composed of frames in which residual bias is still present. As for macrostate 3 (M3), it showcases two basins which appear connected, with an apparent third minima. With such characteristics, it seems to be the most metastable and perhaps the most favorable ensemble for DOAcK. With this in mind, a subsample of 200 members per macrostate was obtained and used for contact analysis; i.e., fraction and concurrence (Figure 9 and Figures S6–S8; respectively). This confirmed that the initial binding mode was indeed lost. One of the most prominent contacts was C285, with a main difference being that in the third macrostate hydrogen bonding is lost in favor of hydrophobic interactions. In contrast, contacts with G284, initially present only in docking with 7WOX, were highly persistent in M3. This is interesting as this residue is often related to PPARγ modulation.

On the other hand, when concurrence of contacts was evaluated, an additional residue is S342, which gains a hydrogen bonding interaction. By comparison, hydrophobic interactions are present with residues near H12. Based on these observations, we confirmed that DOAcK has significant affinity for the LBD in PPARγ. While the exact mechanism for binding remained elusive in our protocol, it is reassuring that there is evidence in favor of modulation within the LBD.

The last step was to assess a qualitative measure for affinity within the LBD. For this, we reparametrized 4JAZ and 7WOX complexes with a more robust force-field and water model. Additionally, we selected a “middle ground” found in PDBID 3VN2, as this structure is co-crystallized with Telmisartan, a known partial modulator of PPARγ. With a similar protocol we found that DOAcK adopts a similar orientation to that of the indole rings in Telmisartan within the LBD (See Figure S5). The role of solvation in the simulations was evaluated by performing protocols both with and without grand canonical Monte Carlo (GCMC).

**Figure 10:**
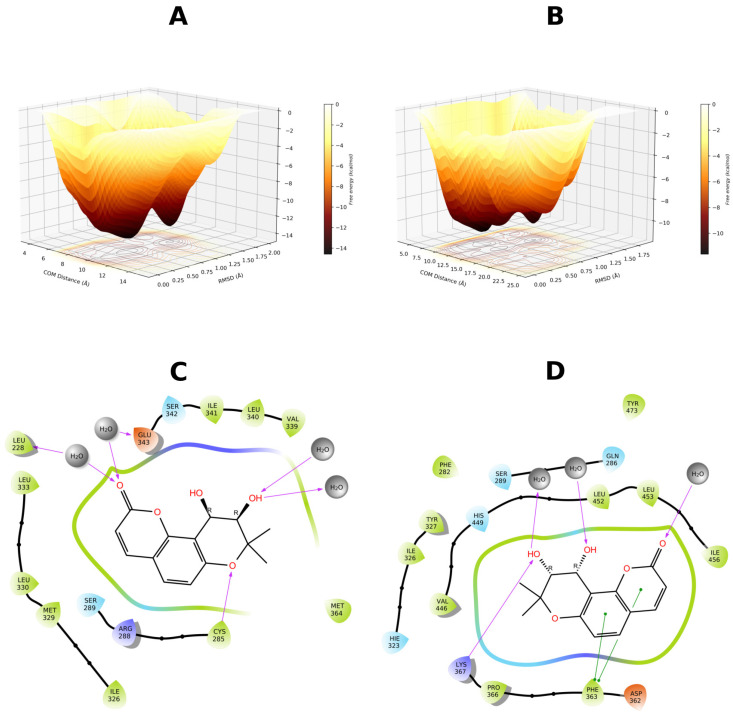
Free energy surface from 500 ns of well-tempered metadynamics for the 4JAZ-DOAcK complex. (**A**) with GCMC solvation; (**B**) without GCMC solvation. A representative pose from within the most prominent basin (**C** & **D** respectively).

Therefore, we present the FES found with 4JAZ (Figure 10). It can be seen that solvation helps to better delimit local minima. Metastability is still present but a higher affinity is found when the pocket is solvated. Notably, water bridges are present in both protocols but these seem to favor R288, as previously found in adaptive sampling. Also, the overall orientation of DOAcK is flipped between metadynamics runs.

**Figure 11:**
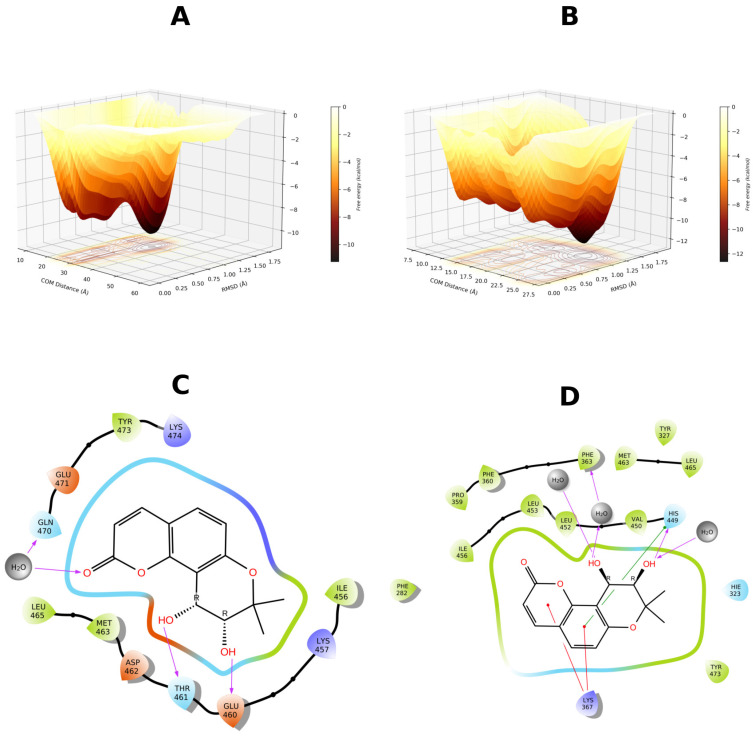
Free energy surface from 500 ns of well-tempered metadynamics for the 3VN2-DOAcK complex. (**A**) with GCMC solvation; (**B**) without GCMC solvation. A representative pose from within the most prominent basin (**C** & **D** respectively).

In contrast, results from 3VN2 exhibit a quite diffuse FES for the GCMC protocol and a very sparse and metastable one for non-GCMC solvation run. (Figure 11). This points to lower affinity when considering the TZD site, as the putative anchoring point for DOAcK. Only the non-GCMC run showed comparable free energy values, a result which also deviates from previous observations.

**Figure 12:**
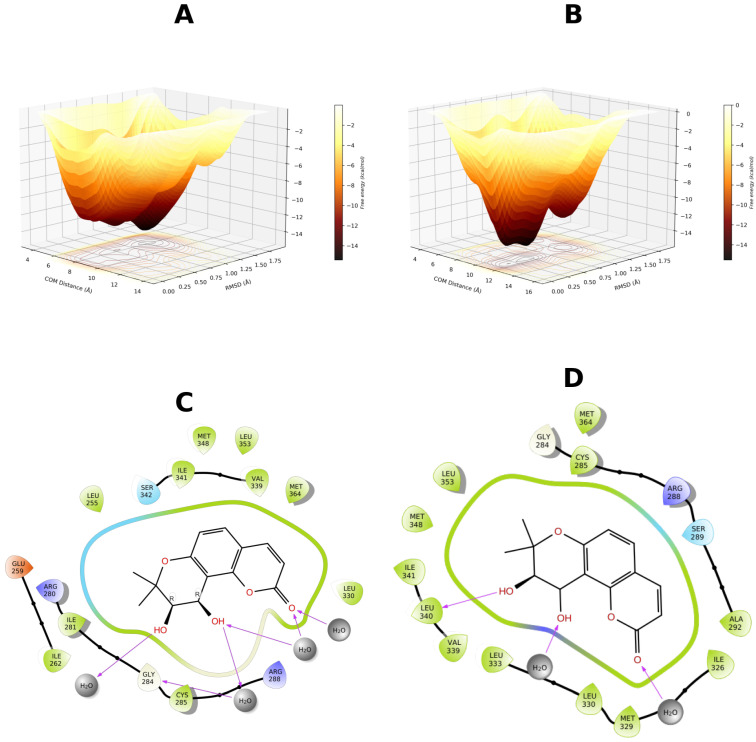
Free energy surface from 500 ns of well-tempered metadynamics for the 7WOX-DOAcK complex. (**A**) with GCMC solvation; (**B**) without GCMC solvation. A representative pose from within the most prominent basin (**C** & **D** respectively).

Finally, for 7WOX-DOAcK complex, well-defined basins were also found (Figure 12). Akin to the previous round, affinity was higher in this complex. Plus, contacts with R288 and S342 were found, consistent with previous observations. Thus, with our molecular modeling approach we can establish that DOAcK showed notable affinity with putative antagonism interactions.

## 4. Discussion

Previous work from our research group demonstrated that extracts obtained from *Arracacia tolucensis* exhibit anti-inflammatory activity [25]. Further phytochemical analyses led to the isolation of Praeruptorin A, an angular pyranocoumarin identified as one of the main specialized metabolites. Subsequent structural modification of this natural product resulted in the synthesis of several derivatives, including DOAcK, a bioactive compound with antidiabetic effects [10].

Taking these findings into consideration, we decided to evaluate the daily administration of DOAcK and its impact on body weight in mice fed a high-fat diet during 10 weeks of the protocol. The mice had free access to food. In the last four weeks the animals which received the treatments Orlistat and DOAcK presented a decrease in body weight (30.59 ± 1.87 and 33.88 ± 0.48 respectively); these values had statistically significance at week number 10 when compared with the HFD group (37.34 ± 1.97) (Figure 1).

It is known that clinical benefits from weight loss appear after 5% of body weight has been reduced, and this decrease is enough to reduce the incidence of diabetes by approximately 50%. Moreover, reaching 5% or more body weight loss is also documented to be beneficial in patients with cardiovascular risk [26].

Pharmacological activities of anti-obesity drugs have diverse mechanisms of action. Examples of these are appetite suppression, inhibition of fat absorption, increased energy expenditure, inhibition of de novo lipogenesis and adipogenesis, among others. Although several drugs are available for obesity management, adverse effects leading to market withdrawal (e.g., rimonabant, sibutramine), short-term usage limitations (e.g., phentermine, diethylpropion), and limited efficacy (e.g., Orlistat) remain major challenges for current pharmacotherapy [27].

Administration of DOAcK was able to reduce about 6.4% of the body weight of the treated group after 4 weeks. C57BL/6 mice are genetically susceptible to developing obesity when fed a high-fat diet [28] and this attribute is useful for evaluating compounds with potential use in obesity management. Several evaluations of natural products in animal models have been described in the literature. Han et al. found administration of chikusetsusaponins decreased about 22% of the body weight of mice with HFD during 9 weeks of protocol [29]. On the other hand, extracts from *Opuntia ficus-indica* decreased about 35% of the body weight of rats treated for 4 weeks [30]. In our current work, administration of DOAcK shows a slight impact on body weight which is comparable to that of aforementioned works, but we performed an experimental protocol where the administration of treatments started at week 6. In that week, all groups with a HFD presented a 50% weight increase above the SD group. The rationale for our protocol is that pharmacological treatment is typically initiated when weight loss is required to improve quality of life, rather than as a palliative measure. Similarly, Kim et al. designed a comparable protocol using rats and found that administration of a crude extract of adlay seeds (*Coix lachrymajobi* var. mayuen) results in a hypolipidemic effect, decreasing body weight and food intake in a significant manner compared with the HFD-control group over 4 weeks of treatment [31]. Therefore, the protocol performed in the current work was helpful in proving the antiobesity effect of DOAcK.

The effect of the treatment on caloric intake may account for the observed reductions in body weight and FBG, but it is evident that other mechanisms are involved.

Dyslipidemia increases the prevalence of NAFLD by 50% [32] and the liver is one of the first organs to suffer damage caused by free fatty acids (FFA). Liver injury occurs when triglyceride accumulation exceeds the storage capacity of hepatocytes, promoting cellular damage that may progress to NAFLD and ultimately to cirrhosis [33]. Controlling the weight of mice with a HFD has been demonstrated to reduce the liver mass on the group treated with ε-polylysine versus the HFD-control group without affecting the energy intake [34]. In the present study, liver mass was significantly reduced. These findings are supported by histological analysis (Figure S6), in which the HFD group exhibited a pale appearance and enlarged hepatocytes, whereas the DOAcK-treated groups showed normal liver morphology. The observed white color and inflammation in the hepatic lobule of the HFD group (Figure S6) is due to excessive accumulation of lipids, which can be considered non-alcoholic steatohepatitis (NASH) by cellular degeneration [35]. This altered architecture was mitigated by administration of DOAcK. These results suggest that this bioactive compound can help in the NAFLD treatment. Moreover, white adipose tissue (WAT) volume was reduced in treated groups versus the HFD group (Figure S7).

Visceral fat accumulation promotes the development of metabolic disorders like glucose intolerance and hyperleptinemia [36]; indeed these dysfunctions are risk factors for NAFLD and diabetes. Prevention of fat accumulation would control the FFA level, WAT volume, NAFLD and body weight. Here, we demonstrated that administration of DOAcK helps in the conditions seen in NAFLD.

To explore the potential mechanisms by which this compound ameliorates these alterations, semi-quantitative endpoint PCR assays were performed to assess the expression of key genes involved in NAFLD. These results indicate that DOAcK maintains IL-6 levels in WAT comparable to those of the healthy group (Figure 3). Concurrently, in the liver, DOAcK significantly decreased the expression of PPARγ (Figure 4). PPARγ is mainly expressed in WAT, but it is also present in other tissues such as the liver, as it is related to lipid storage [37]. Decreasing the expression of PPARγ has been demonstrated to possess an effect on 3T3-L1 cells, preventing the adipocyte differentiation which reduces glucose and triglycerides levels [8]. Furthermore, reduced expression of PPARγ has been associated with decreased WAT mass and reduced hepatic steatosis in mice fed an HFD [38]. In this sense, in our work, DOAcK decreased the expression of PPARγ in both WAT and liver, which suggests that this mixture could have consequences in absorption and elimination of FFA in the system. Consistent with these findings, histological analysis of the HFD group revealed hepatocytes with balloon-like morphology, whereas DOAcK-treated groups exhibited morphology comparable to that of the control group (Figure S6).

On the other hand, in obese and diabetic patients IL-6 levels are elevated [39]; conversely to what is expected, this cytokine is high during exercise [40]. Previous reports suggest that IL-6 plays a key role in the regulation of energy metabolism, as IL-6-deficient mice develop obesity accompanied by elevated leptin levels and fasting blood glucose [41]. Moreover, mice lacking IL-6 had a reduced lipid metabolism [42]. Although there is not a definitive consensus regarding the IL-6 role in obesity, some recent works have demonstrated IL-6 has beneficial effects regardless of its primary role as an inflammatory mediator. Cheng et al. showed that IL-6 promotes insulin secretion and this effect is altered during obesity. They concluded reactivation of the IL-6 pathway could be of potential interest for type 2 diabetes [43]. Similarly, Trinh et al. showed that upon blocking the IL-6 receptors, an impaired fat mobilization pathway occurs [44]. Peppler et al. demonstrated that administration of IL-6 has a beneficial effect over hepatic glucose and insulin homeostasis [45]. These works support the idea that IL-6 has a catabolic function in fat metabolism.

In this work, we found that mice treated with DOAcK showed IL6 levels close to the control group. Wallenius et al. suggest IL-6 exerts an effect at central level, decreasing food intake and increasing energy expenditure. We think this effect by DOAcK helps to preserve the level of FBG and energy intake (Figure 2) [41].

PPARγ is a transcription factor that belongs to the nuclear receptor superfamily that regulates gene expression by recruiting co-regulators that can be classified as either coactivator or corepressor. Some relevant structural sites that make up this protein are a ligand binding domain composed of 13 alpha helices (H1-H12 and H2′) and, within this structure, a y-shaped internal ligand binding pocket (LBP) as well as an activation function 2-surface (AF-2) made up of H3-5 and H12 [46,47].

Given that this nuclear receptor can either act as repressor or as promoter on the regulation of genes related to lipid and carbohydrate metabolism as well as cell proliferation, apoptosis, and angiogenesis, it is important to note that this difference is dependent on the type of ligands it recruits, as this modulates how AF-2 can be found, either on an active conformation that favors coactivator binding, or contrastingly in an inactive state, favoring corepressor binding [48].

There are many types of ligands that can interact with this pocket; furthermore, the responses they can elicit range from full or partial agonism to antagonism and inverse agonism. This poses a problem, as these intricate mechanisms need to be simplified to some degree. A common starting point is ligand-based similarity, whose main premise is that similar chemotypes tend to share similar biological activities [49]. It follows that structure selection is based on reference ligands. However, given the structural and conformational diversity of PPARγ, it presses for alternative approaches. For instance, it has been suggested that docking results are improved when spatial similarities exist [50]. Thus, to evaluate PPARγ modulation, the sole use of a given PDB structure may prove fruitless. For this reason, we attempted ensemble modeling, as it has been shown that free energy landscapes are highly dependent from starting conformations [51].

In the PPAR case, it is well known that H12 plays a fundamental role in PPARγ dynamics as it can take on different conformations that may impede the recruitment of any co-regulators [52,53,54]. In lieu of this, the most discussed interactions by which full agonists are believed to act in this manner is by forming a key hydrogen bond with residue Y473 present in H12. Importantly, said bond has not been typically observed for partial agonists. Antagonists, on the other hand, make unfavorable interactions with residues on H2 and therefore do not stabilize H12.

Regarding the presented protocol, docking scores showed an average value of −77.0 which by itself is not very significant. This is due to the overarching difficulty of ranking docking results, as these often rely on scoring functions with varying degrees of success [55]. A further issue is the added difficulty of scarce enantiomeric discrimination solely by means of docking [56]. Nevertheless, it may suggest a favorable environment for DOAcK interaction-wise within the LBD, which is the main rationale of the consensus approaches that we presented herein. For instance, well-tempered metadynamics have proven robust for discrimination of true binders from ‘decoys’ [24].

Our proposal is that DOAcK modulates PPARγ dynamics, as it seems to bind between H2 and H3 and persist there by means of hydrophobic interactions and perhaps water bridges. It has been suggested that most partial agonists exhibit contacts with C285 and R288 [57]. Considering the sole role of interactions, it would seem that DOAcK would be some sort of agonist; yet, when accounting for the experimental findings herein, this does not seem to be the case.

We hypothesize that DOAcK stays within the LBD, preventing co-binding mechanisms which result in antagonistic activity. Similar behavior has been reported for betulinic acid [47] and dithiothreitol [58]. Additionally, HL005, a well-known PPARγ antagonist, was found to make hydrophobic contacts with C285, I341, M348 and M364 [59]; all of these were found consistently by our adaptive sampling of DOAcK.

Pocket solvation is often an overlooked issue in drug binding assessment due to its inherent complexity [60]. It is interesting that water molecules seem to provide an additional layer of anchoring for DOAcK, a consistent result even in some runs without GCMC solvation. Nonetheless, similar to prior observations [61], proper water sampling significantly improved FES resolution and the overall consistency of free energy values between runs.

Additionally, H2 and H3 are linked via a highly mobile region known as the omega loop, sensitive to dynamics. It has been remarked how this loop can affect the proper regulation of some genes related to obesity and diabetes by mediating the cyclin-dependent kinase 5 (Cdk5) phosphorylation of PPARγ at residue S245 [23]. Another important aspect regarding the omega loop dynamics is a tripartite salt bridge between the adjacent H6-7 loop and H11-12 loop, as a disruption within this interaction heavily destabilizes AF-2 surface disordering the active H12 conformation [52].

Interestingly, as important as the omega loop is for PPARγ dynamics, it is often overlooked as it is difficult to resolve its structure given its high mobility.

## 5. Conclusions

In this work, we confirmed the effect of DOAcK to control fasting blood glucose and body weight, alongside its efficacy in terms of improving dangerous conditions associated with obesity such as Non-Alcoholic Fatty Liver Disease and white adipose tissue hypertrophy. Taken together with our previous work, these findings suggest that DOAcK could be an important bioactive compound to treat metabolic syndrome. The results of this study suggest that DOAcK may modulate the activity of PPARγ, potentially contributing to reductions in body weight, increased expression of IL-6, and partial restoration of hepatic and white adipose tissue morphology. Additionally, the proposed binding mode of DOAcK to PPARγ supports a possible mechanistic basis for these effects. Overall, these findings provide preliminary evidence supporting the potential of DOAcK as a candidate for Non-Alcoholic Fatty Liver Disease, warranting further investigation.

## Figures and Tables

**Table 1:** Recorded organ weight after administration of DOAcK HFD-fed mice.

Organ	SD	HFD	HFD + Orlistat	HFD + DOAcK
Liver (g) g/bw	1.06 ± 0.0437 * 0.02 ± 0.0017	1.634 ± 0.1757 0.049 ± 0.0064	0.947 ± 0.0985 * 0.063 ± 0.0079	1.089 ± 0.0417 * 0.032 ± 0.0014 *
Kidney (g) g/bw	0.329 ± 0.0100 * 0.013 ± 0.0004 *	0.594 ± 0.0125 0.018 ± 0.0017	0.350 ± 0.0169 * 0.012 ± 0.0005 *	0.441 ± 0.0097 * 0.013 ± 0.0002 *
Spleen (g) g/bw	0.060 ± 0.0029 * 0.002 ± 0.0001 *	0.123 ± 0.0153 0.004 ± 0.0003	0.074 ± 0.0083 * 0.003 ± 0.0002 *	0.051 ± 0.0514 * 0.002 ± 0.0001 *
Fat pad (g) g/bw	0.350 ± 0.0317 * 0.014 ± 0.0012 *	1.983 ± 0.2204 0.057 ± 0.0059	1.745 ± 0.3453 0.054 ± 0.0089	1.407 ± 0.1322 0.042 ± 0.0039
Pancreas (g) g/bw	0.125 ± 0.0132 0.005 ± 0.0006	0.146 ± 0.0128 0.004 ± 0.0003	0.150 ± 0.0145 0.004 ± 0.0002	0.101 ± 0.0054 * 0.003 ± 0.0002 *

g/bw: specific organ weight in grams over body weight. * Statistically significant versus HFD group, one-way ANOVA, comparison by Dunnett’s test (n = 8, mean ± SEM, *p* < 0.05).

## Data Availability

The original contributions presented in this study are included in the article/Supplementary Material. Further inquiries can be directed to the corresponding authors.

## Supplementary Materials

The following supporting information can be downloaded at: https://www.mdpi.com/article/10.3390/biom16050724/s1, Table S1: Primer sequences used for PCR assay; Figure S1: Scoring variation as obtained from ten independent replicas of OBPMD; Figure S2: Comparison between SR11023 and DOcK binding modes in PDBID 6C5T. Poses from docking are presented for DOAcK (top, gold; selected, violet); Figure S3: Comparison between Telmisartan and DOAcK binding modes in PDBID 3VN2; Figure S4: Lag time validation based on implied timescales (ITS) as obtained from Time Independent Component Analysis (TICA); Figure S5: Spectral analysis and timescale separation of Time Independent Components (TICs); Figure S6: Contact concurrence as obtained from adaptive sampling and PCCA, for Macrostate 1; Figure S7: Contact concurrence as obtained from adaptive sampling and PCCA, for Macrostate 2; Figure S8: Contact concurrence as obtained from adaptive sampling and PCCA, for Macrostate 3; Figure S9: Histological analysis of hepatic tissue; Figure S10: Histological analysis of WAT; Figure S11: Original electrophoresis images.

## Abbreviations

The following abbreviations are used in this manuscript:

DOAcK	3′,4′-Di-*O*-acetyl-*cis*-khellactone
ee	Enantiomeric excess
FBG	Fasting blood glucose
FFA	Free fatty acids
GCMC	Grand canonical Monte Carlo
NAFLD	Non-alcoholic fatty liver disease
HFD	High-fat diet
LBD	Ligand-binding domain
SD	Standard diet
