# Supplementary material for: Evaluation of 3′,4′-Di-O-acetyl-cis-khellactone as a Putative Antagonist of PPARγ Using Experimental and Computational Modeling
Source: Biomolecules. 2026 May 14;16(5):724. doi: 10.3390/biom16050724 (PMC13204854; doi:10.3390/biom16050724)
Supplement: Supplementary file 1 [file biomolecules-16-00724-s001.zip › biomolecules-4285569-supplementary.pdf]

Supplementary Material

Evaluation of 3′,4′-di-*O*-acetyl-*cis*-khellactone as a putative antagonist of PPARγ using experimental and computational modeling

Elix Alberto Domínguez-Mendoza[1,*], Fernando Daniel Prieto-Martínez[2,*], Yelzyn Galván-Ciprés[3], Eleuterio Burgueño-Tapia[3] and Cynthia Ordaz-Pichardo[4]

Table S1. Primer sequences used for PCR assay

Genes	Primer sequences 5′ to 3′
β-actin Forward	GGACTCCTATGTGGGTGACGAG
β-actin Reverse	CTTCTCCATGTCGTCCCAGTTGG
PPARγ Forward	GCTGAATCACCCAGAGTCCTCTC
PPARγ Reverse	GCTCTGTGACGATCTGCCTGAG
IL-6 Forward	GCCTTCTTGGGACTGATGCTG
IL-6 Reverse	CAGGTCTGTTGGGAGTGGTATC

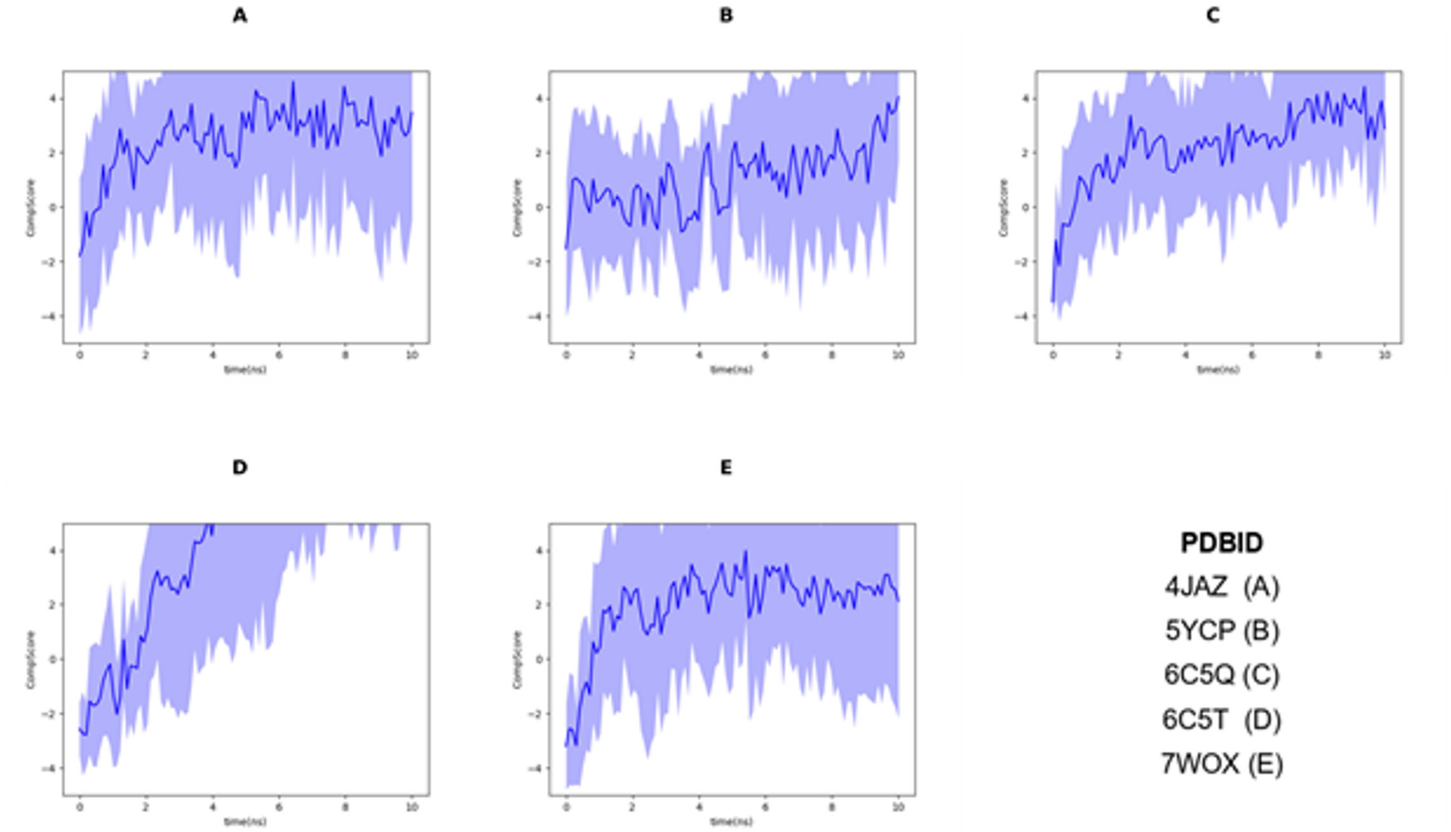

Figure S1. Scoring variation as obtained from ten independent replicas of OBPMD.

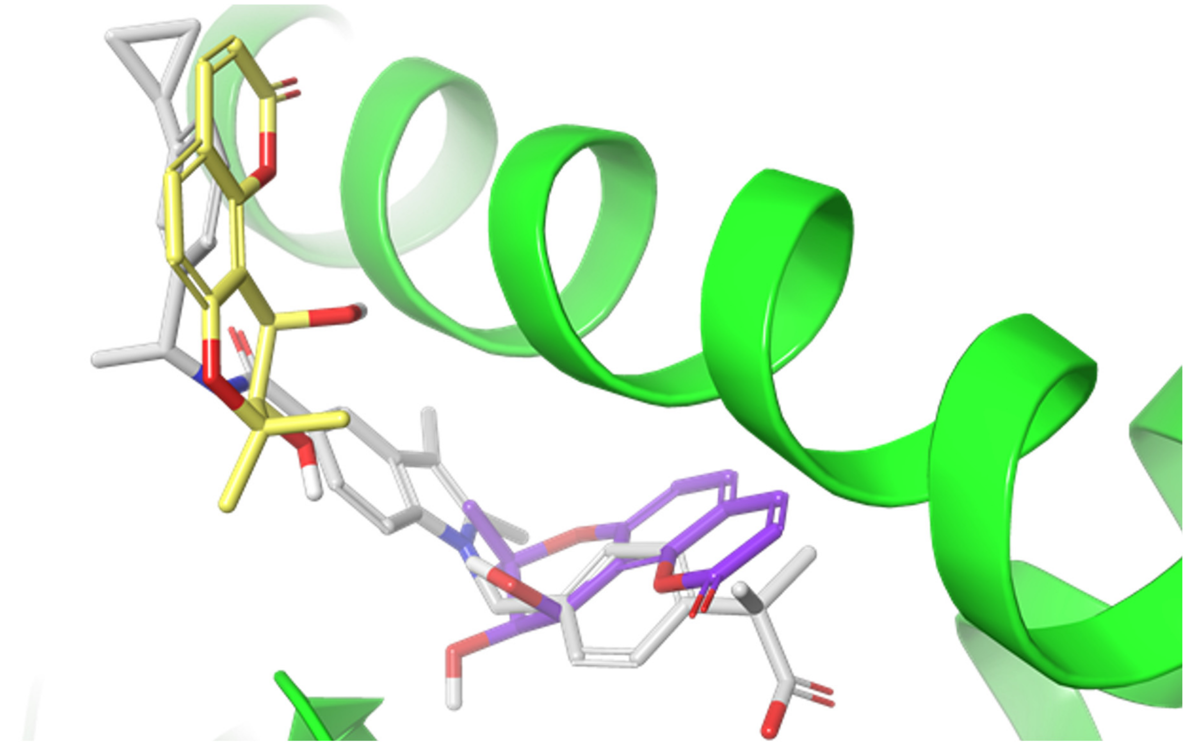

Figure S2. Comparison between SR11023 and DOcK binding modes in PDBID 6C5T. Poses from docking are presented for DOAcK (top, gold; selected, violet).

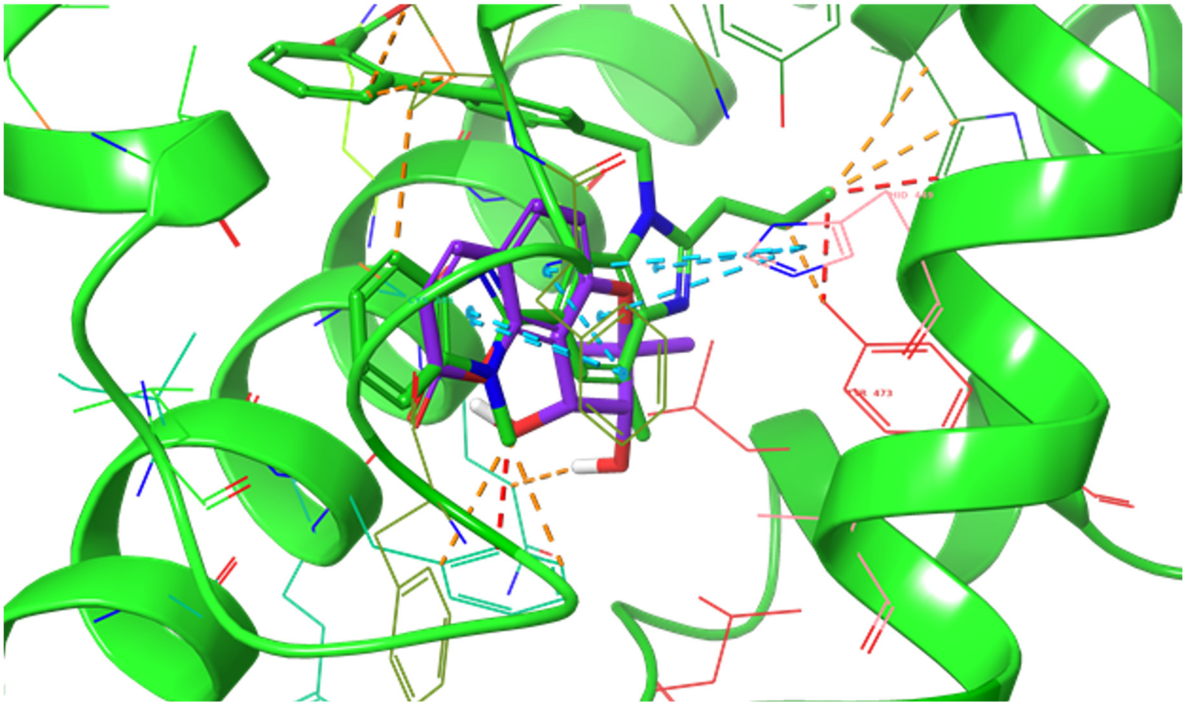

Figure S3. Comparison between Telmisartan and DOAcK binding modes in PDBID 3VN2.

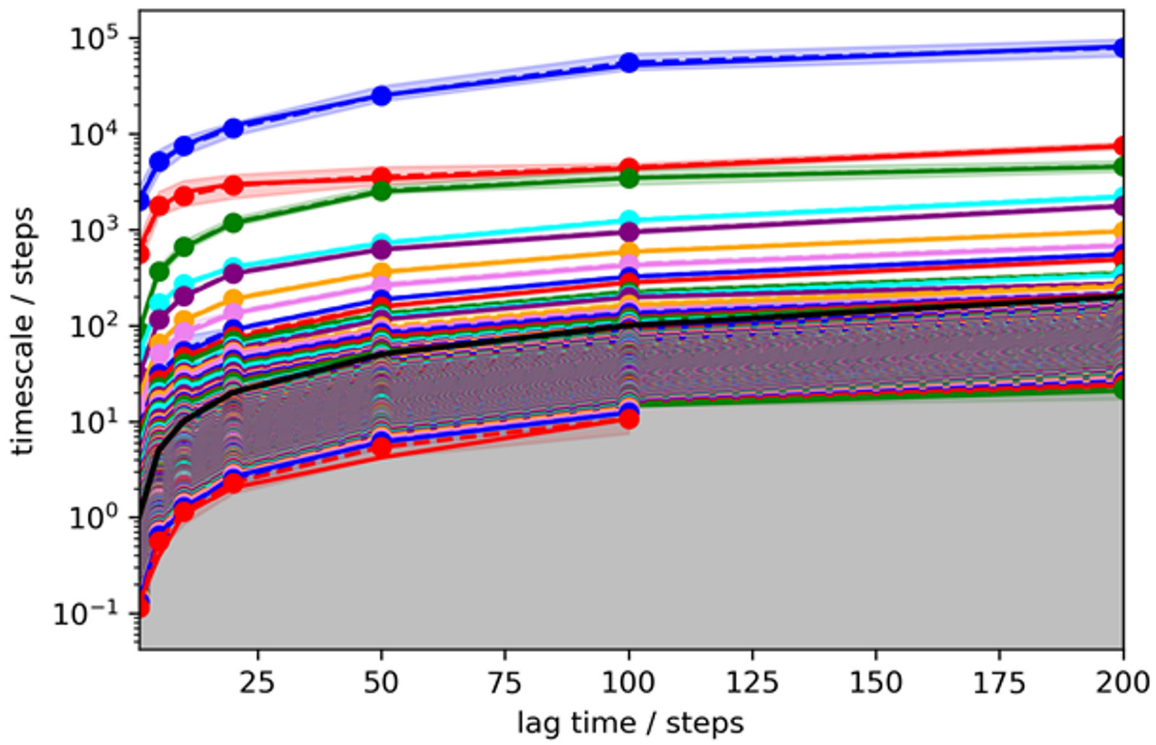

Figure S4. Lag time validation based on implied timescales (ITS) as obtained from Time Independent Component Analysis (TICA).

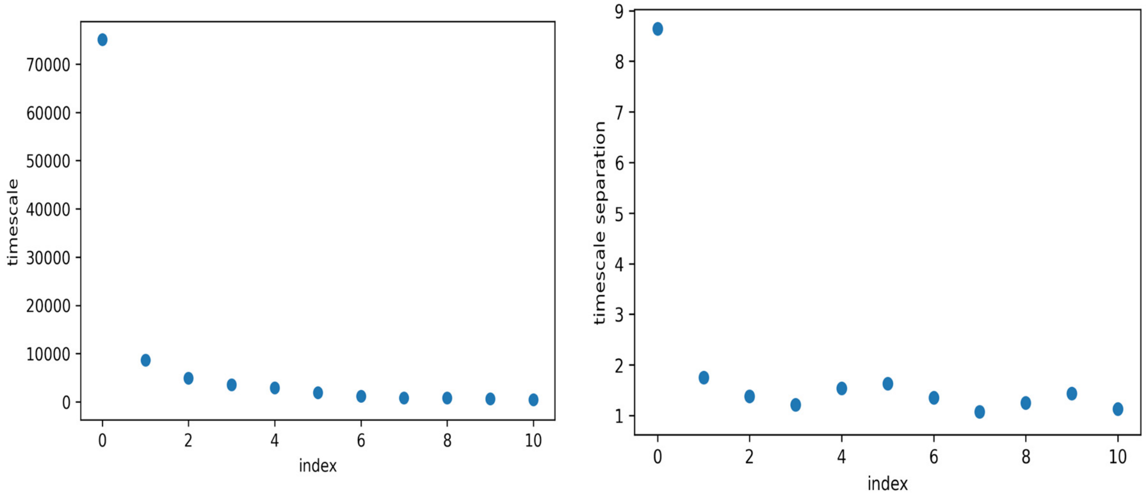

Figure S5. Spectral analysis and timescale separation of Time Independent Components (TICs).

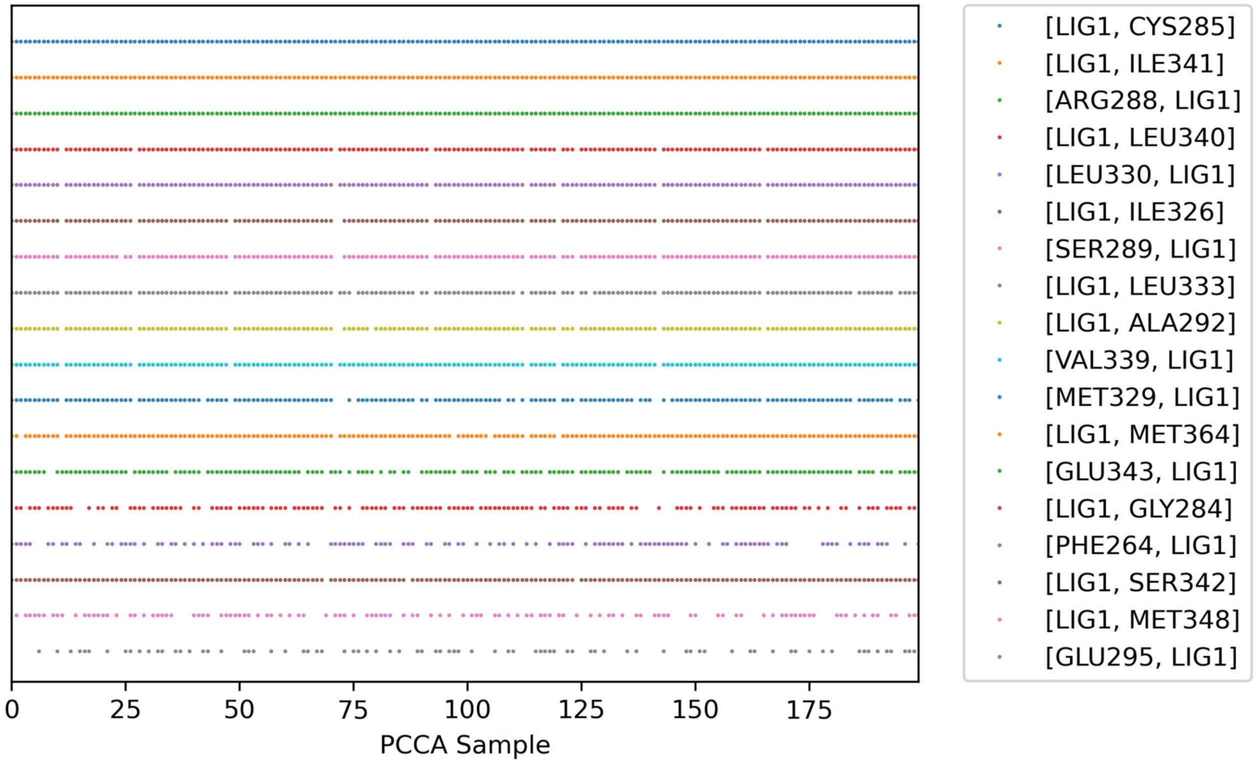

Figure S6. Contact concurrence as obtained from adaptive sampling and PCCA, for Macrostate 1.

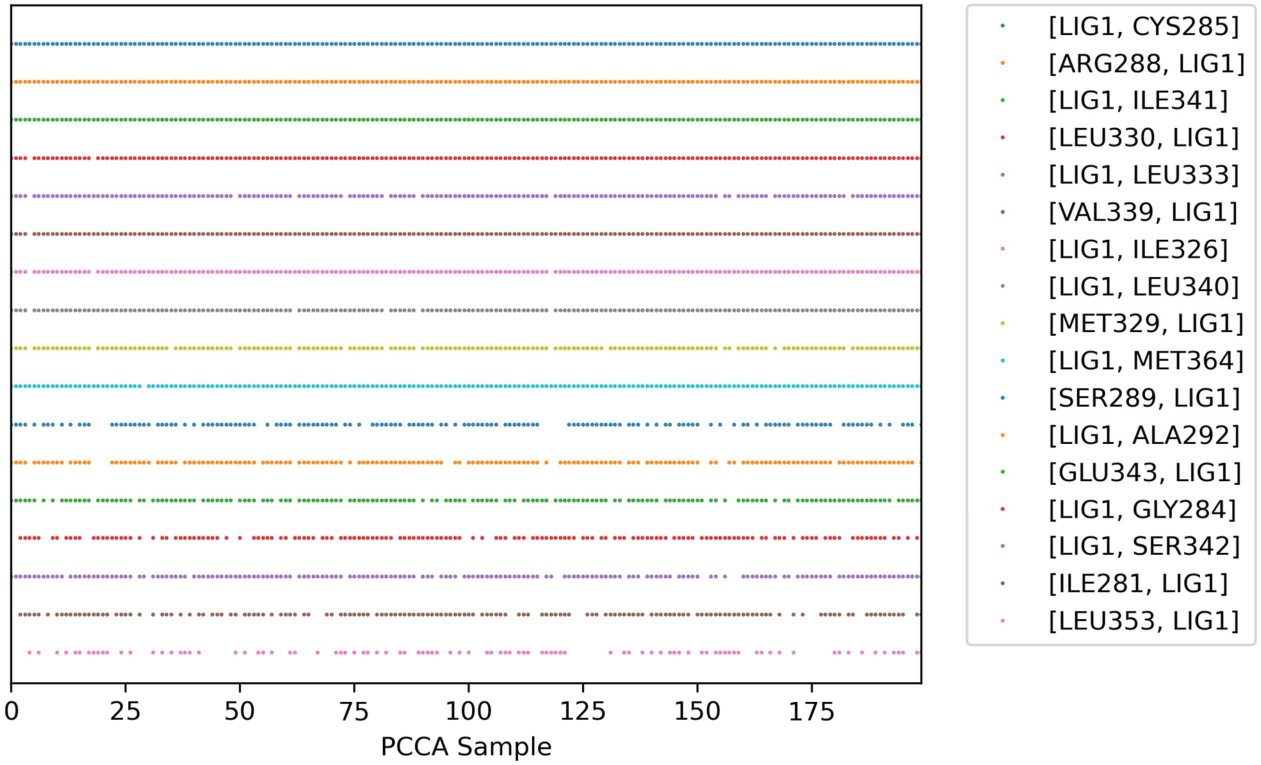

Figure S7. Contact concurrence as obtained from adaptive sampling and PCCA, for Macrostate 2.

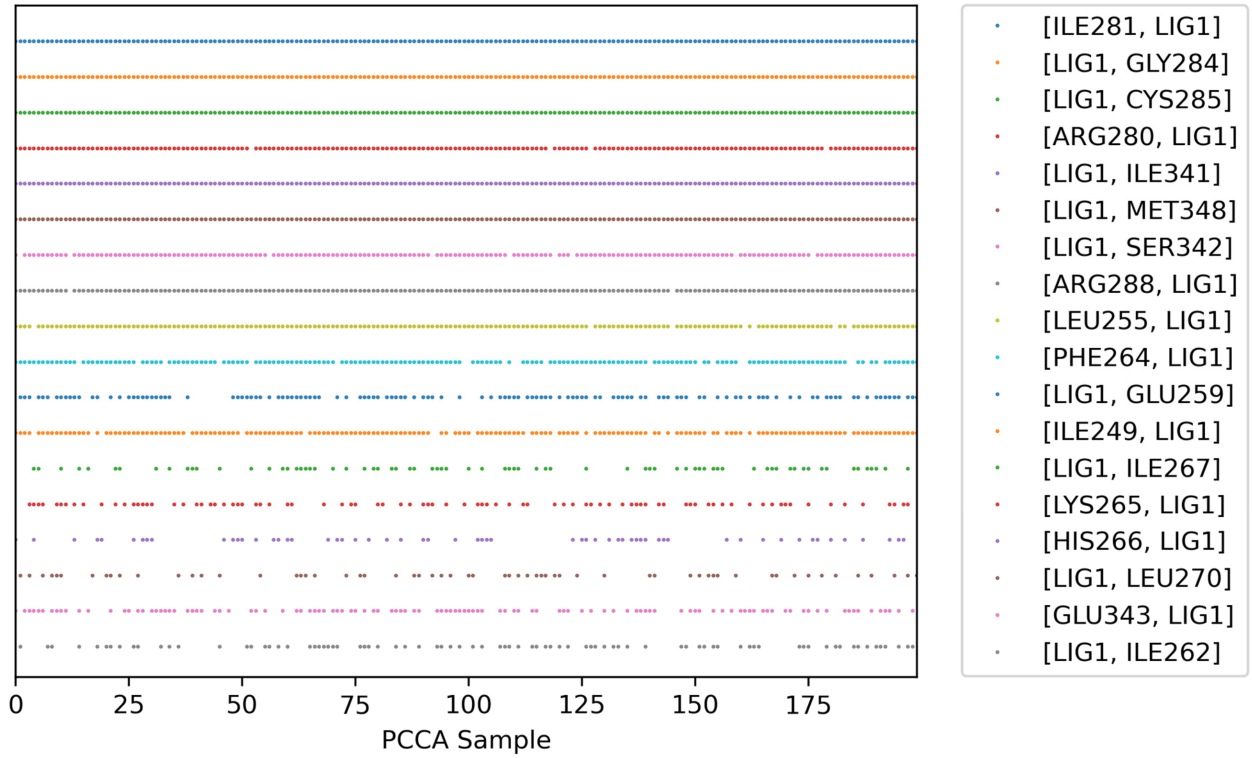

Figure S8. Contact concurrence as obtained from adaptive sampling and PCCA, for Macrostate 3.

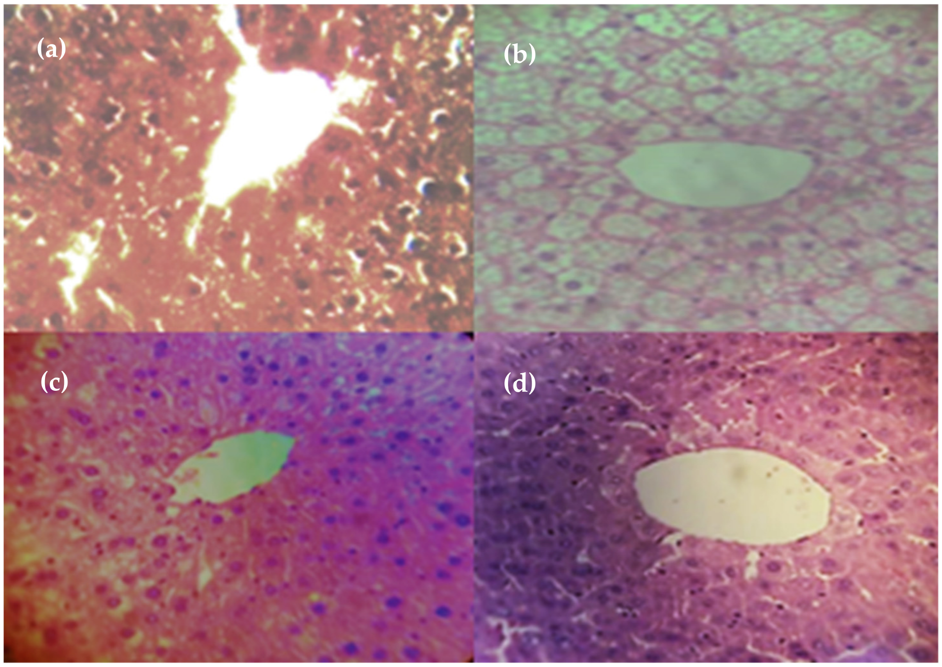

Figure S9. Histological analysis of hepatic tissue: (**a**) SD; (**b**) HFD; (**c**) HFD + Orlistat; (**d**) HFD + DOAcK

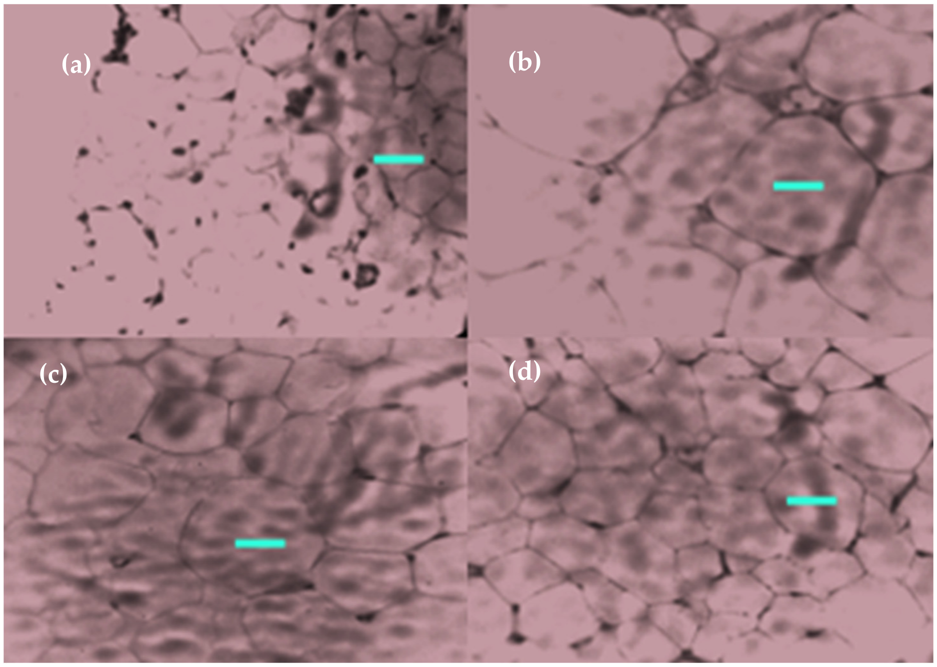

Figure S10. Histological analysis of WAT: (**a**) SD; (**b**) HFD; (**c**) HFD + Orlistat; (**d**) HFD + DOAcK

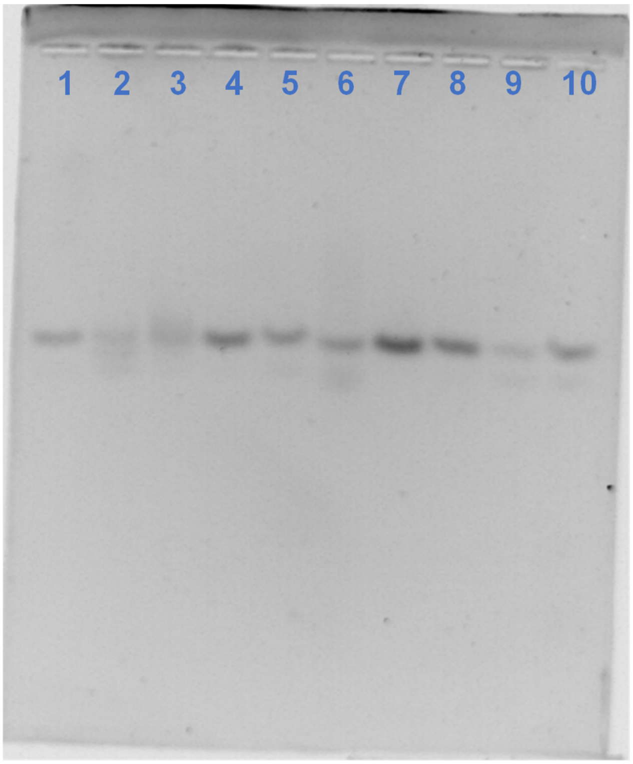

a) IL6: 1) SD-WAT 2) HFD-WAT 3) Vehicle-WAT 4) Orlistat-WAT 5) DOAcK-WAT 6) SD-Liver 7) HFD-Liver 8) Vehicle-Liver 9) Orlistat-Liver 10) DOAcK-Liver

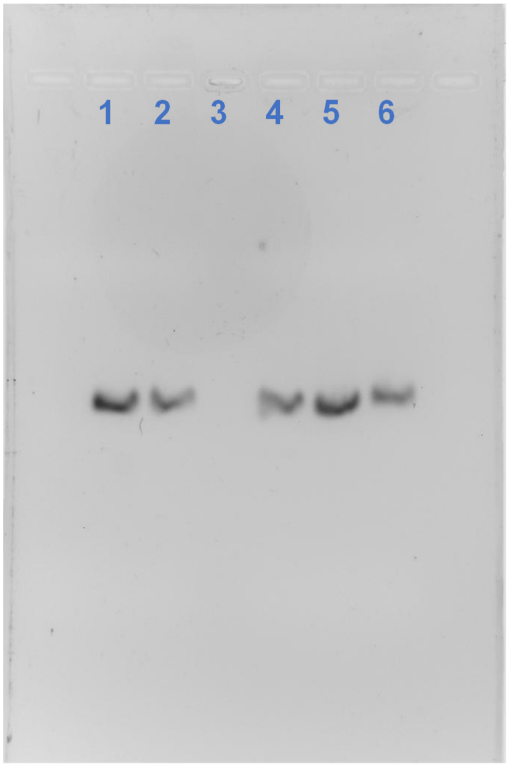

a) PPARγ in white adipose tissue: 1) SD 2) HFD 3) Not loaded 4) Vehicle 5) Orlistat 6) DOAcK

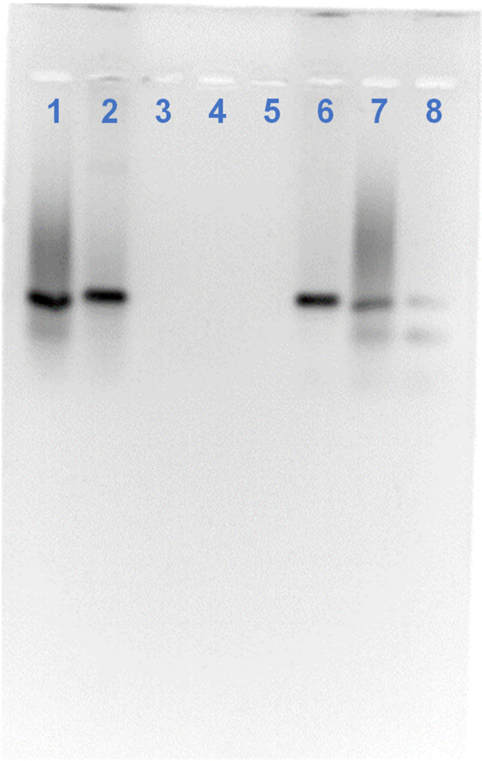

b) PPARγ in liver: 1) SD 2) HFD 3) Not loaded 4) Not loaded 5) Not loaded 6) Vehicle 7) Orlistat 8) DOAcK

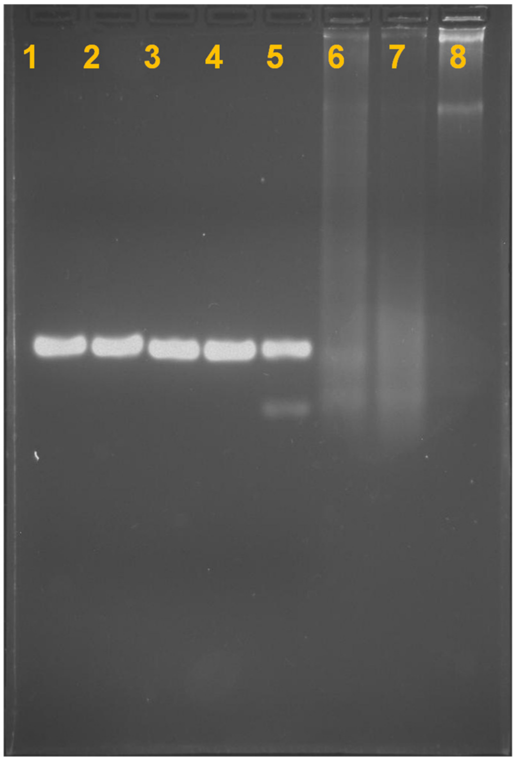

c) Actin in white adipose tissue: 1) SD 2) HFD 3) Vehicle 4) Orlistat 5) DOAcK

e)

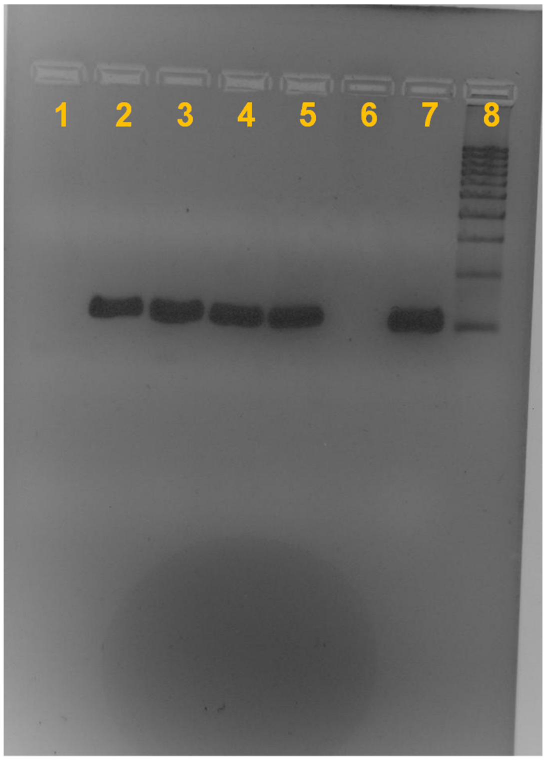

d) Actin in liver: 1) Not loaded 2) SD 2) HFD 3) Vehicle 4) Orlistat 5) Not loaded 6) DOAcK

f)

Figure S11. Original electrophoresis images
